# Supplementary material for: OnionNet-2: A Convolutional Neural Network Model for Predicting Protein-Ligand Binding Affinity Based on Residue-Atom Contacting Shells
Source: Front Chem. 2021 Oct 27;9:753002. doi: 10.3389/fchem.2021.753002 (PMC8579074; doi:10.3389/fchem.2021.753002)
Supplement: Supplementary file 1 [file DataSheet1.PDF]

Supplementary Material

1 THE STATISTICAL INFORMATION OF THE TRAINING SET AND VALIDATING SET WHEN USING DIFFERENT VERSIONS OF PDBBIND DATABASE.

Generally, the size of the datasets has an impact on the predictive ability of the DL model.Sun et al. (2017); Lavecchia (2019) To make a fair comparison with previous scoring functions, we re-trained the model with two elder versions (v.2016 and v.2018) of the PDBbind database, and the number of samples in the training set and validating set is shown in Table S1. In addition, we only re-trained the models using N = 58, 60 and 62. The performance on core set v.2013 and core set v.2016 are summarized in Fig S1. It is clear that, although the three versions of PDBbind database differed greatly in size, the R values of our re-trained models are almost the same. This suggests that the difference between these three databases (in detail, PDBbind database v.2016, v.2018 and v.2019) has a rather limited impact on our OnionNet-2 model.

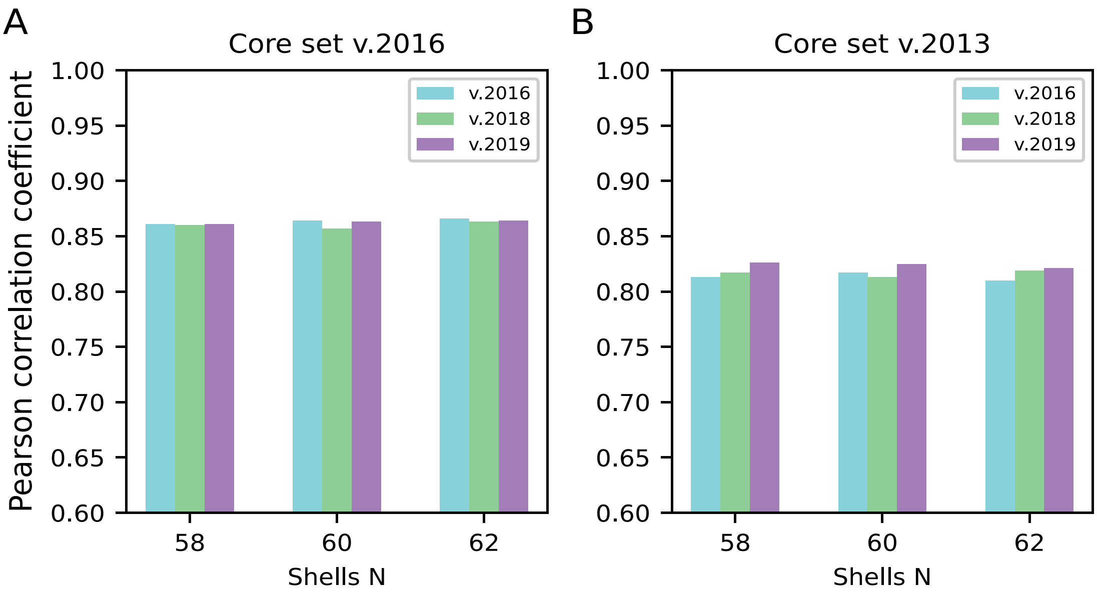

Figure S1. Pearson correlation coefficients achieved by OnionNet-2 on (A) core set v.2016 and (B) core set v.2013 when different versions of PDBbind database are used for training (the total number of shells is set to 58, 60 and 62).

Table S1. The number of samples in training set and validating set for three versions of PDBbind database.

	Training set	Validating set
v.2016	11820	1000
v.2018	14686	1000
v.2019	16626	1000

2 COMPARISON OF ONIONNET-2 WITH SOME REPRESENTATIVE SCORING FUNCTIONS.

Table S2. Comparison of the predictive power of scoring functions on the core set v.2016 and v.2013

	CASF-2016		CASF-2013	
scoring functions	R	RMSE	R	RMSE
OnionNet-2	0.864	1.164	0.821	1.29
AGLNguyen and Wei (2019)	0.833	1.271	0.792	1.45
K_{deep}Jiménez et al. (2018)	0.82	1.27		
OnionNetZheng et al. (2019)	0.816	1.278	0.78	1.45
RF-Score-v3Stepniewska-Dziubinska et al. (2018)	0.80	1.39	0.74	1.51
PafnucyStepniewska-Dziubinska et al. (2018)	0.78	1.42	0.70	1.61
kNN-ScoreKhamis and Gomaa (2015)			0.672	1.65
X-ScoreKhamis and Gomaa (2015)			0.614	1.78
ChemScoreKhamis and Gomaa (2015)			0.592	1.82
Autodock VinaGaillard (2018)			0.54	1.90
AutodockGaillard (2018)			0.54	1.91

3 THE RESULT ACHIEVED BY ONIONNET-2 ON CSAR-HIQ SUBSETS.

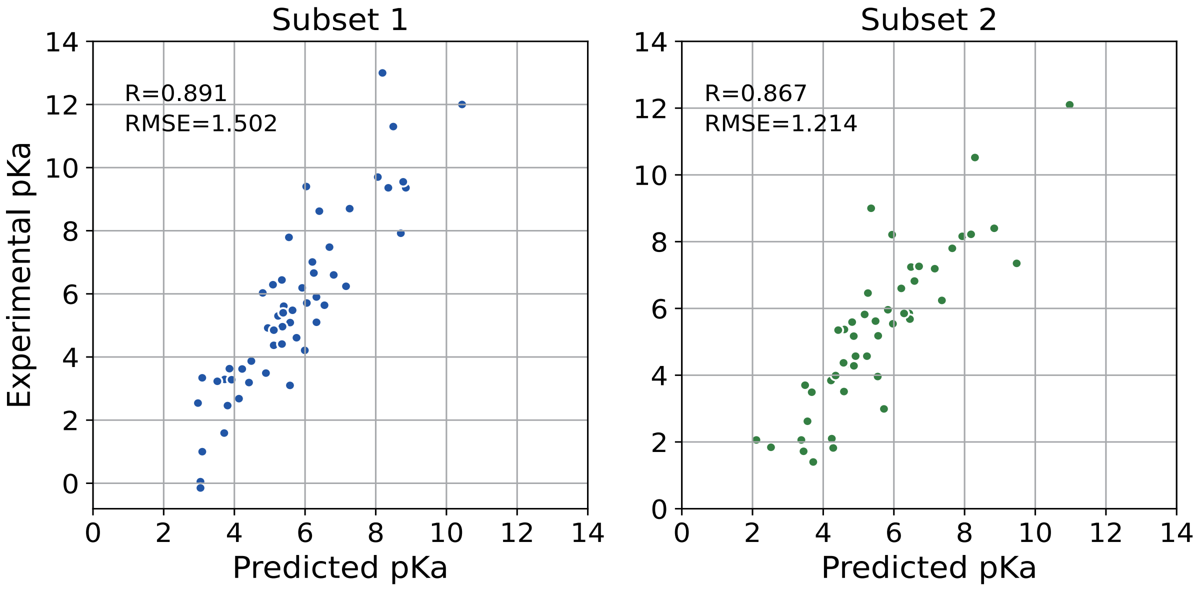

Figure S2. The result achieved by OnionNet-2 on CSAR-HiQ subsets.

4 THE DETAILED INFORMATION OF THE GENERATION OF DECOYS.

In this study, we used AutoDock Vina to generate non-native binding pose (called decoys). The sampling space is a 27 Å × 27 Å × 27 Å cubic, which is centered on the geometric center of the native ligand binding pose. The exhaustiveness value was set to 12. It is worth noting that AutoDock Vina ignores the effects of water molecules and ions when calculating protein-ligand binding energy. In addition, changes in the ligand binding poses will be accompanied by changes in the surrounding microenvironment. Therefore, the receptor-decoy complexes here do not include water molecules and ions.

5 ONIONNET-2 PREDICTED PKA WITH RESPECT TO EXPERIMENTAL DETERMINED PKA ON THREE SERIES SUBSETS.

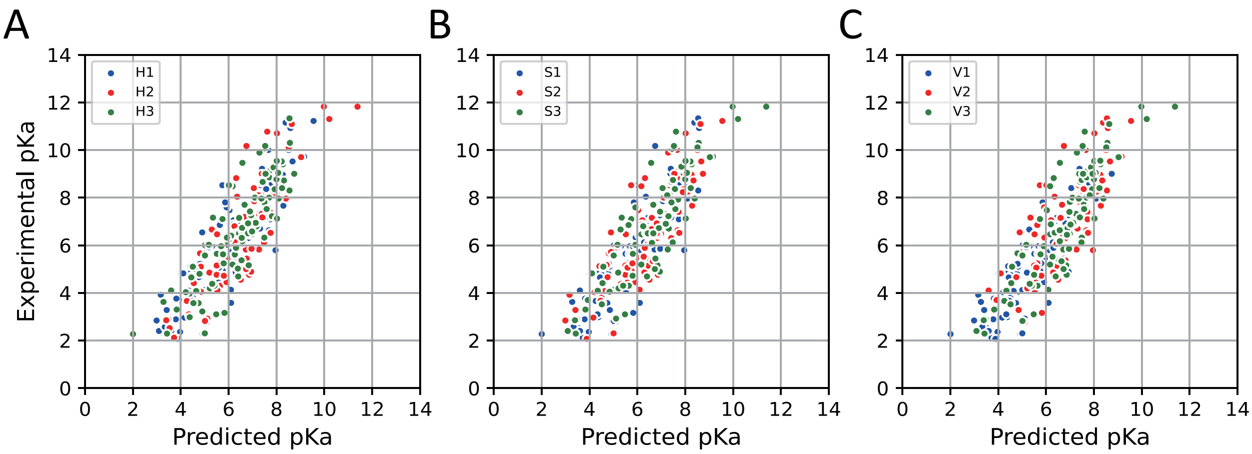

Figure S3. OnionNet-2 predicted pKa with respect to experimental determined pKa on (A) H-, (B) S- and (C) V-series subsets.

REFERENCES

Gaillard, T. (2018). Evaluation of autodock and autodock vina on the casf-2013 benchmark. *Journal of chemical information and modeling* 58, 1697–1706

Jiménez, J., Skalic, M., Martinez-Rosell, G., and De Fabritiis, G. (2018). K deep: protein–ligand absolute binding affinity prediction via 3d-convolutional neural networks. *Journal of chemical information and modeling* 58, 287–296

Khamis, M. A. and Gomaa, W. (2015). Comparative assessment of machine-learning scoring functions on pdbbind 2013. *Engineering Applications of Artificial Intelligence* 45, 136–151

Lavecchia, A. (2019). Deep learning in drug discovery: opportunities, challenges and future prospects. *Drug discovery today* 24, 2017–2032

Nguyen, D. D. and Wei, G.-W. (2019). Agl-score: Algebraic graph learning score for protein–ligand binding scoring, ranking, docking, and screening. *Journal of chemical information and modeling* 59, 3291–3304

Stepniewska-Dziubinska, M. M., Zielenkiewicz, P., and Siedlecki, P. (2018). Development and evaluation of a deep learning model for protein–ligand binding affinity prediction. *Bioinformatics* 34, 3666–3674

Sun, C., Shrivastava, A., Singh, S., and Gupta, A. (2017). Revisiting unreasonable effectiveness of data in deep learning era. In *Proceedings of the IEEE international conference on computer vision*. 843–852

Zheng, L., Fan, J., and Mu, Y. (2019). Onionnet: a multiple-layer intermolecular-contact-based convolutional neural network for protein–ligand binding affinity prediction. *ACS omega* 4, 15956–15965
